# Comparative transcriptome analysis of axillary buds in response to the shoot branching regulators gibberellin A3 and 6-benzyladenine in *Jatropha curcas*

**DOI:** 10.1038/s41598-017-11588-0

**Published:** 2017-09-12

**Authors:** Jun Ni, Mei-Li Zhao, Mao-Sheng Chen, Bang-Zhen Pan, Yan-Bin Tao, Zeng-Fu Xu

**Affiliations:** 1 Key Laboratory of Tropical Plant Resources and Sustainable Use, Xishuangbanna Tropical Botanical Garden, Chinese Academy of Sciences, Menglun, Mengla, Yunnan 666303 China; 2 Institute of Technical Biology & Agriculture Engineering, Hefei Institutes of Physical Science, Chinese Academy of Sciences, Hefei, Anhui 230031 China; 3 College of Life Sciences, University of Chinese Academy of Sciences, Beijing, 100049 China

## Abstract

Cytokinin (CK) is the primary hormone that positively regulates axillary bud outgrowth. However, in many woody plants, such as *Jatropha curcas*, gibberellin (GA) also promotes shoot branching. The molecular mechanisms underlying GA and CK interaction in the regulation of bud outgrowth in *Jatropha* remain unclear. To determine how young axillary buds respond to GA_3_ and 6-benzyladenine (BA), we performed a comparative transcriptome analysis of the young axillary buds of *Jatropha* seedlings treated with GA_3_ or BA. Two hundred and fifty genes were identified to be co-regulated in response to GA_3_ or BA. Seven *NAC* family members were down-regulated after treatment with both GA_3_ and BA, whereas these genes were up-regulated after treatment with the shoot branching inhibitor strigolactone. The expressions of the cell cycle genes *CDC6*, *CDC45* and *GRF5* were up-regulated after treatment with both GA_3_ and BA, suggesting they may promote bud outgrowth via regulation of the cell cycle machinery. In the axillary buds, BA significantly increased the expression of GA biosynthesis genes *JcGA20oxs* and *JcGA3ox1*, and down-regulated the expression of GA degradation genes *JcGA2oxs*. Overall, the comprehensive transcriptome data set provides novel insight into the responses of young axillary buds to GA and CK.

## Introduction

The plant above-ground architecture is primarily due to the complex spatial-temporal regulation of shoot branching. After axillary bud formation, the bud can undergo immediate outgrowth or become dormant or quiescent^1–3^. Axillary bud activity is closely correlated with various endogenous and environmental stimuli, such as phytohormones, light, and nutrients^4^. Among these environmental signals, light is a major factor that affects plant development^5^. Plants can modulate shoot branching to obtain maximum light radiation for increased production of photosynthetic products, primarily sucrose. In pea, sucrose itself has been implicated in the regulation of bud outgrowth^6^. The endogenous phytohormones levels are, in part, a reflection of the growth conditions that plants are subjected to^7^. Two types of hormones are involved in the regulation of bud outgrowth: cytokinin (CK), the major hormone promoting bud outgrowth^8^, and auxin and strigolactones (SLs), both of which inhibit bud outgrowth^9–11^. However, at the molecular level, how these hormones regulate bud outgrowth remains elusive. *BRANCHED1* (*BRC1*), which encodes a transcription factor, inhibits axillary bud outgrowth in many species (e.g., pea, *Arabidopsis* and rice)^12–14^. In pea and *Arabidopsis*, *BRC1* expression is negatively regulated by CK but induced by GR24 (a synthetic analog of SL)^14–16^. However, *BRC1* levels are not affected by GR24 treatment in rice^13^.

Auxins are traditionally a group of phytohormones involved in the regulation of growth and development of plants, the efflux of which is important for axillary bud outgrowth^8^. Thus far, most studies have focused on the interactions of auxins, CKs and SLs in the regulation of bud outgrowth^1^. Other hormones, such as gibberellins (GAs), were also considered to be involved in the regulation of bud outgrowth, although its effects in different species vary a lot^4^. In rice and *Arabidopsis*, GA plays a negative role in controlling shoot branching^17, 18^. Recently, we observed that in many perennial woody plants, such as *Jatropha curcas* and papaya, GA is a positive regulator of axillary bud outgrowth^19^. In sweet cherry, GA treatment also promoted shoot branching in the spring after bud break^20^. In hybrid aspen, GA biosynthesis is crucial in axillary bud formation and activation^2^. These findings demonstrated that in many perennial woody plants, the regulation of shoot branching may differ from that in annual, herbaceous plants, such as *Arabidopsis*, pea and rice. The involvement of GAs complicates the regulatory network of axillary bud outgrowth. Nevertheless, the molecular mechanisms of how GAs, through interactions with CK, regulate shoot branching in *Jatropha* remain elusive.

In recent years, numerous studies on various aspects of the biofuel woody plant *J. curcas* have been reported, including seed development^21^, seed oil biosynthesis^22^, seed toxicity^23, 24^, flower development^25^ and adaptations to biotic and abiotic stresses^26–29^, genome and transcriptome studies^30–35^ and shoot branching governed by different phytohormones^19, 36^. In *J. curcas*, GA and CK are positive regulators of shoot branching and corporately regulate the axillary bud outgrowth in antagonism with some other hormones, such as SLs and auxin^19^. Immediate bud outgrowth can be detected a few days after gibberellin A3 (GA_3_) or 6-benzyladenine (BA, a synthetic CK) treatment. Quantitative real-time PCR analyses have shown that GA_3_ and BA have common target genes, such as *JcBRC1*, *JcBRC2* and *JcMAX2* ^19^, key inhibitors of bud outgrowth in pea, rice and *Arabidopsis* ^12, 16, 37–42^. These results suggest that the downstream regulatory networks of bud outgrowth mediated by these two hormones could interact in *J. curcas* ^19^.

Transcriptome sequences generated using high-throughput sequencing techniques are efficient and data-rich^43^. High-throughput sequencing is an effective method to identify the candidate genes or pathways involved in the regulation of different biological processes at the transcriptome level. To advance the current understanding of how the young axillary buds respond to exogenously applied GA_3_ or BA in *J*. *curcas*, we performed transcriptome analyses of the nodal stem after mock, GA_3_ and BA treatments. Subsequent comparisons of the global expression profiles of mock-, GA_3_- or BA-treated samples enabled the identification of many GA_3_- and BA-responsive genes. Specifically, the identification of the common target genes of these two hormones would help in selecting candidates involved in the regulation of axillary bud outgrowth in *J*. *curcas*. Further detailed analyses of the function of these genes could provide novel insights into the regulatory network of phytohormones that control axillary bud outgrowth.

## Results and Discussion

### Identification of differentially expressed genes (DEGs) in response to GA_3_ and BA

We previously reported that exogenous treatment with GA_3_ or BA effectively promote the axillary bud outgrowth in *J. curcas* (Fig. S1), by suppressing negative regulators of shoot branching, such as *JcBRC1*, *JcBRC2*, and *JcMAX2* ^19^. To obtain further insights into the genetic regulation of the young axillary buds response to GA_3_ or BA at a genome-wide level, we performed an RNA sequencing-based transcriptome analysis of *Jatropha* young axillary buds exposed to GA_3_ or BA treatment. Then, we identified the candidate genes involved in the regulation of the axillary bud outgrowth in *J. curcas*. Approximately 12 million clean reads per sample (biological replicate) were obtained from the mock, GA_3_ and BA experimental groups (Table S1). A plot showing the relationships among 9 samples from the three groups (mock, GA_3_ and BA) was generated based on multidimensional scaling through the “plotMDS.dge” function of the edgeR package^44^ (Fig. S2). The results clearly showed the nine samples were well separated into three groups, indicating the good repeatability of the RNA-seq results. Together with the relative high value of Q30 and high percentage of mapped reads from the nine samples of the three groups (Table S1), these analysis suggested that the RNA-seq result is qualified for further downstream analysis.

**Figure 1:**
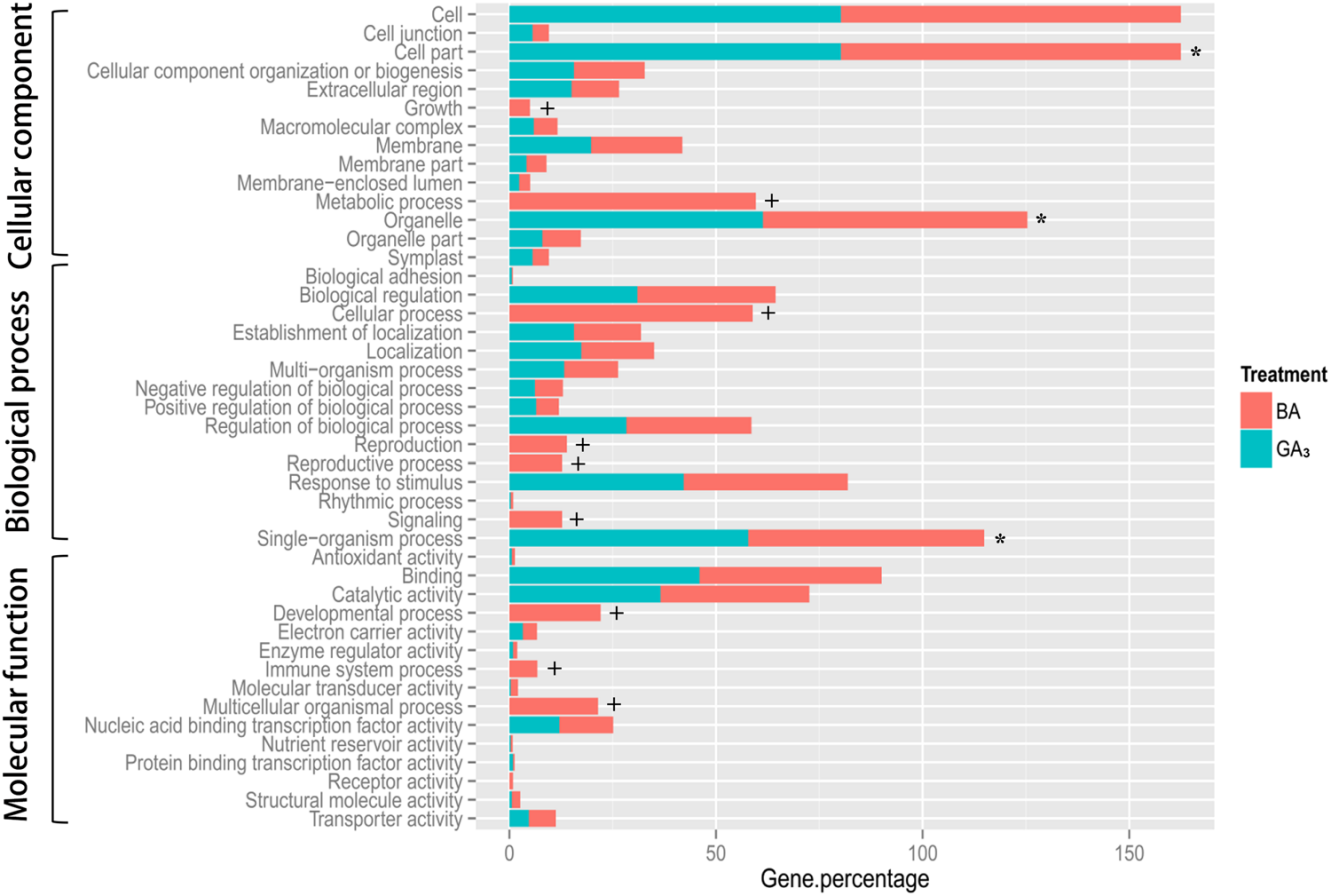
GO analysis of the DEGs by GA_3_ or BA treatment. DEGs were assigned to three categories: cellular component, biological process and molecular function. The most abundant terms were marked with “*”; BA-specific GO terms were marked with “+”. Gene percentage was calculated as: number of DEGs in one GO term/number of total analyzed DEGs.

Transcriptome analysis showed that 1365 genes were down-regulated and 1357 genes were up-regulated after GA_3_ treatment, whereas 2619 genes were down-regulated and 2728 genes were up-regulated after BA treatment (false discovery rate (FDR) < 0.05) (Fig. S3), implying that BA is a more general regulator than GA_3_ in the axillary bud outgrowth in *Jatropha* seedlings. The differentially expressed genes (DEGs) with a threshold of fold change ≥ 2 and FDR value ≤ 0.05, including 1141 DEGs from BA treatment (Table S2) and 429 DEGs from GA_3_ treatment (Table S3), were chosen for further analysis. Gene Ontology (GO) annotation showed that the DEGs of GA_3_ or BA treatment were predominantly classified in the “cell part”, “organelle” and “single-organism process” categories (Fig. 1). However, many of the DEGs belonging to the “metabolic process”, “cellular process”, “multicellular organismal process”, “reproduction”, “reproductive process”, “developmental process”, “signaling” and “growth” categories were present after BA treatment but not after GA_3_ treatment (Fig. 1, and Table S4).

**Figure 2:**
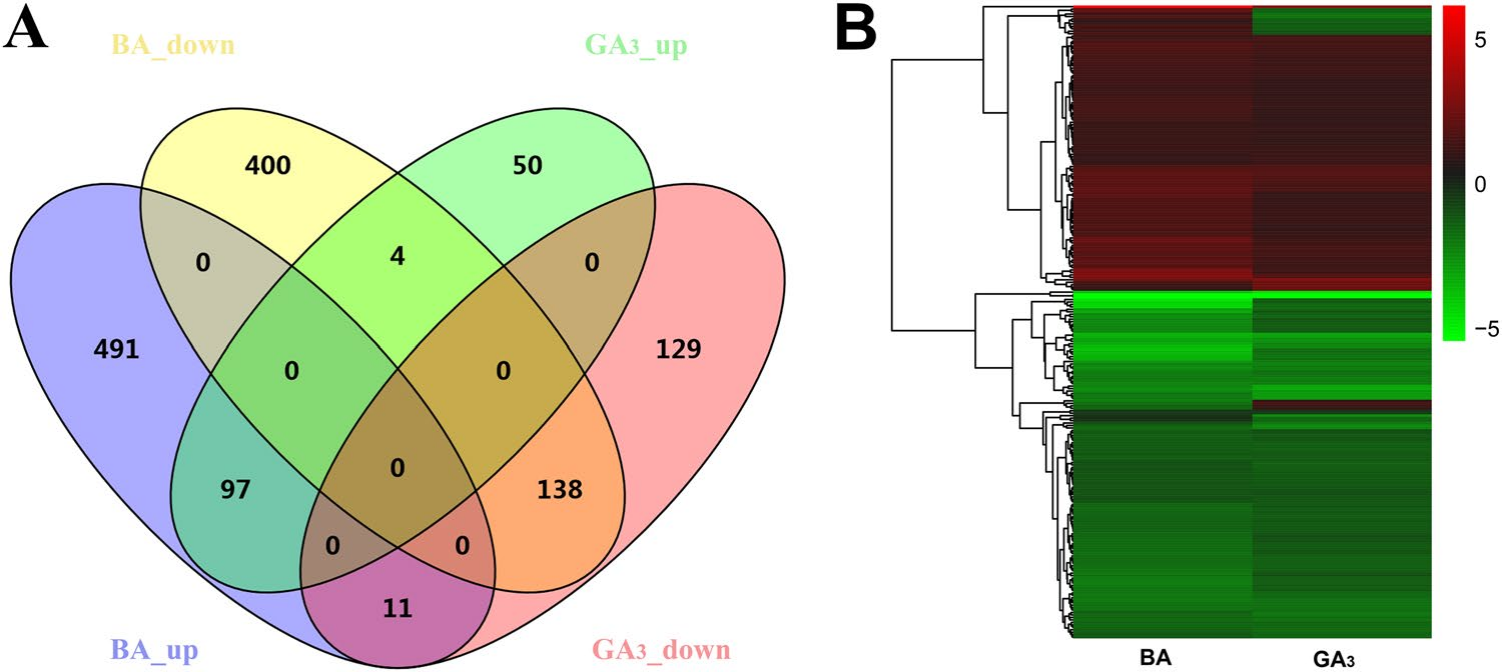
Venn diagram (**A**) and heat map clustering (**B**) of the co-regulated genes by GA_3_ and BA treatment. The input information was listed in Table S5. TMM-normalized fragments per kilobase of transcript per million fragments sequenced (FPKM) values were log-transformed prior to clustering.

**Figure 3:**
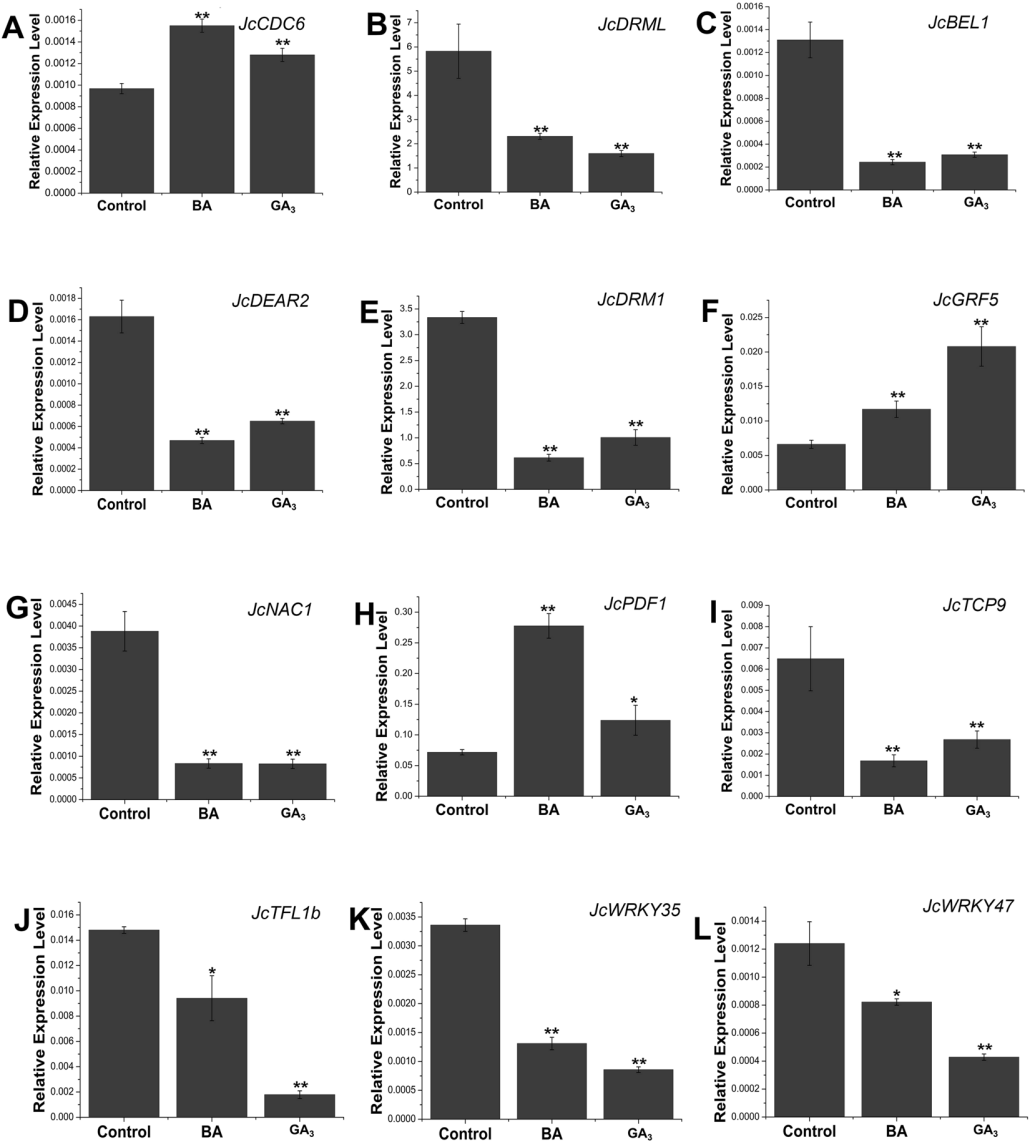
Quantitative real-time PCR analysis of the transcription factors regulated by both GA_3_ and BA treatment. The expression of *JcCDC6* (**A**, Cluster-2004.0), *JcDRML* (**B**, Cluster-26.12832), *JcBEL1* (**C**, Cluster-26.17792), *JcDEAR2* (**D**, Cluster-26.17120), *JcDRM1* (**E**, Cluster-26.5833), *JcGRF5* (**F**, Cluster-26.21078), *JcNAC1* (**G**, Cluster-26.19073), *JcPDF1* (**H**, Cluster-26.15852), *JcTCP9* (**I**, Cluster-26.19926), *JcTFL1b* (**J**, Cluster-26.17723), *JcWRKY35* (**K**, Cluster-26.10011) and *JcWRKY47* (**L**, Cluster-1476.0) was analyzed in the buds of *Jatropha* seedlings at 12 h. *GAPDH* was used as the internal reference. The error bars represent SE (n = 3). Student’s t-test was used to determine significant differences between the indicated and control groups. Significance levels: *P < 0.05; **P < 0.01.

**Figure 4:**
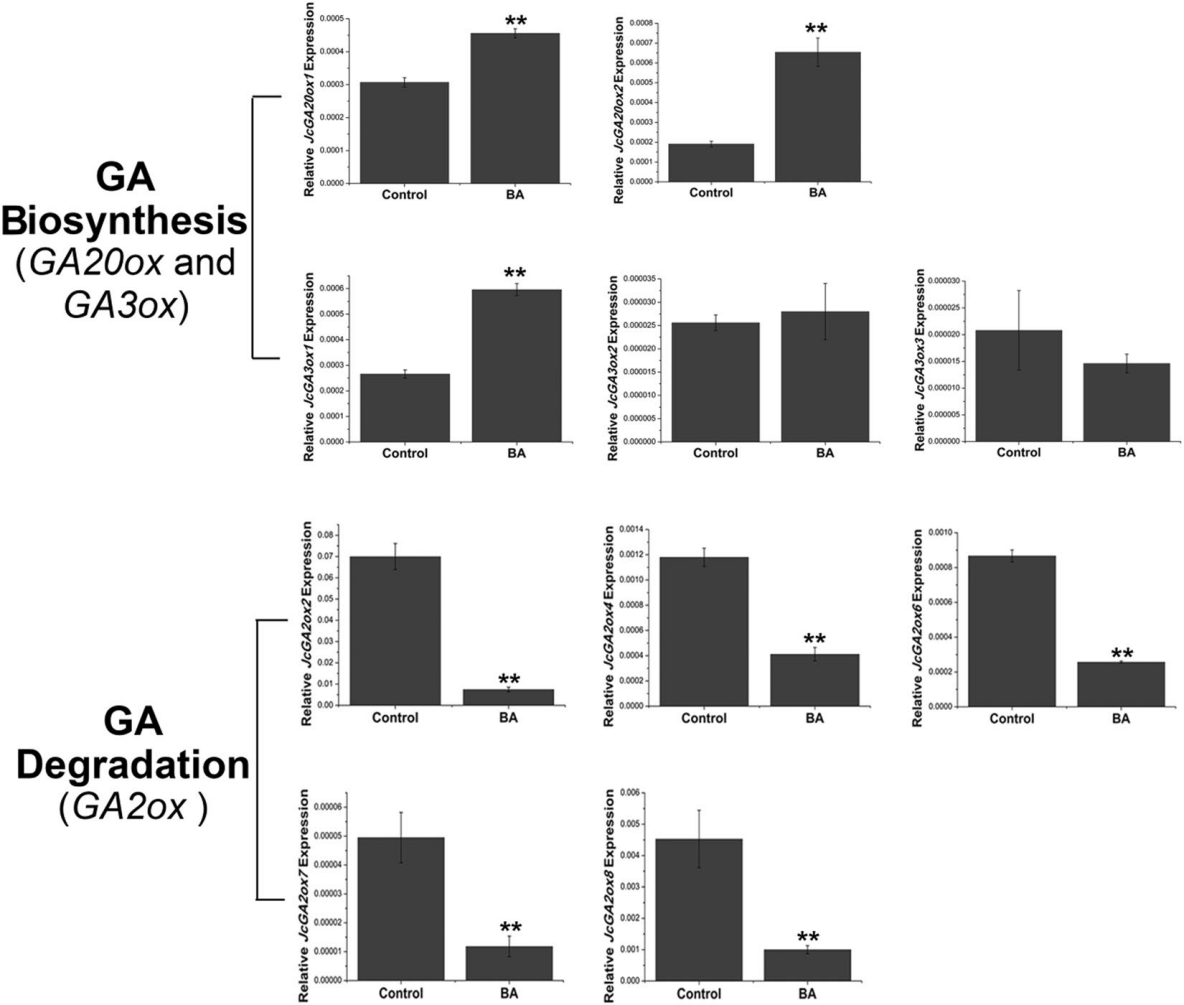
BA regulates GA metabolism in the buds. BA promoted the expression of the GA biosynthesis genes *JcGA20ox1*, *JcGA20ox2* and *JcGA3ox1*, and simultaneously inhibited the expression of all GA degradation genes *JcGA2ox*s in *J. curcas*. Cluster IDs: *JcGA20ox1*, Cluster-26.319; *JcGA20ox2*, Cluster-26.16883; *JcGA3ox1*, Cluster-26.16438; *JcGA3ox2*, Cluster-26.14433; *JcGA3ox3*, Cluster-26.14518; *JcGA2ox2*, Cluster-26.9482; *JcGA2ox4*, Cluster-26.18818; *JcGA2ox6*, Cluster-26.14996; *JcGA2ox7*, Cluster-26.16004; *JcGA2ox8*, Cluster-26.18801. *GAPDH* was used as the internal reference. The error bars represent SE (n = 3). Student’s t-test was used to determine the significant differences between the indicated and control groups. Significance levels: **P < 0.01.

Among the DEGs identified in this study, there are 891 DEGs specifically regulated by BA, 179 DEGs by GA_3_, and 250 co-regulated genes that responded to both GA_3_ and BA (Fig. 2A and Tables S5–S7). The expression clustering analysis of the 250 co-regulated genes showed that most of these genes had similar expression patterns in the GA_3_- and BA-treated groups (Fig. 2B). Among the 250 co-regulated DEGs, a total of 97 genes were up-regulated, and 138 genes were down-regulated in both the GA_3_- and BA-treated groups, and only 15 genes were differentially expressed in these two groups (Fig. 2A and Table S5). The GO analysis showed the most abundant GO terms of the 97 co-up-regulated DEGs were “compound/protein binding”, “metabolic process” and “biosynthetic process”, indicating the accelerated cell activity during the axillary bud outgrowth (Table S8). In contrast, the 138 co-down-regulated DEGs were mostly distributed in response pathways, such as “response to stress”, “response to chemical”, and “response to endogenous stimulus” (Table S9), which may result from a trade-off between active growth and defense to biotic and abiotic stress^45, 46^. Among the 15 differentially co-regulated DEGs (Table S5), three were identified to encode enzymes of GA biosynthesis (*JcGA20ox2*, Cluster-26.16883) and degradation (*JcGA2ox2*, Cluster-26.9482 and *JcGA2ox4*, Cluster-26.18818), and two are GA-responsive genes *JcGASA6* (GA-regulated family protein, Cluster-26.3799)^47^ and *JcBG3* (1,3-β-glucanase 3, Cluster-545.0), whose homologs in hybrid aspen were involved in axillary bud dormancy release and branching^2, 48^. To validate the expression profiles of these co-regulated genes, we selected 12 transcription factors, whose homologs are involved in the regulation of shoot branching (*JcBEL1*, *JcDRM1*, *JcDRML*, and *JcTFL1b*) and cell division (*JcCDC6*, *JcGRF5*, and *JcNAC1*), or belong to the well characterized families of plant transcription factors (*JcDEAR2*, *JcPDF1*, *JcTCP9*, *JcWRKY35*, and *JcWRKY47*), for qPCR analysis. The results were consistent with the transcriptome data (Fig. 3, Table S5). In addition, the different expression profiles of the GA biosynthesis gene *GA20ox2* (Cluster-26.16883) and the GA degradation genes *GA2ox2* (Cluster-26.9482) and *GA2ox4* (Cluster-26.18818) between GA_3_ and BA treatment, which were revealed by the transcriptome data (Table S5), were also confirmed by qPCR analyses (Figs 4 and S4). Taken together, at the transcriptome level, these results demonstrated that bud outgrowth in *J. curcas* could be cooperatively regulated by GA_3_ and BA.

### Bud outgrowth and dormancy-related genes are commonly regulated by GA_3_ or BA

Most dormant axillary bud cells in the annual plants *Arabidopsis* or pea are believed to be blocked in G_1_ phase, and the cell cycle can be reactivated concomitantly with the initiation of bud outgrowth^49–51^. Cell division control (CDC) proteins, such as CDC6 and CDC45, are important regulators of the cell cycle that are ubiquitously found in animals, plants and fungi^52–58^. After GA_3_ or BA treatment, the expression of *JcCDC6* (Cluster-2004.0) and *JcCDC45* (Cluster-26.14626) was significantly increased (Fig. 3A and Table S5). The expressions of three genes encoding *DNA primase-polymerase* (*JcPrimPol*, Cluster-26.12318), *DNA polymerase alpha subunit B* (*JcPOLA2*, Cluster-609.2) and the catalytic subunit of DNA polymerase α (*JcICU2*, Cluster-761.0) were up-regulated by both GA_3_ and BA treatment (Table S5). Moreover, the expression of another important plant growth regulator *GRF5* (Cluster-26.21078), which is involved in the regulation of cell proliferation in the leaf primordia^59^, was also increased in the axillary bud after GA_3_ or BA treatment (Fig. 3F and Table S5). These results suggested that bud outgrowth promoted by GA_3_ or BA in *J. curcas* may be due, in part, to triggering the cell cycle machinery, which could be one of the key points where different phytohormones convergently regulate initiation of the axillary bud outgrowth.

Two other genes, *BELL1* (*BEL1*) and *TERMINAL FLOWER 1* (*TFL1*), are both potential negative regulators of shoot branching in *Arabidopsis* ^59–61^. In sorghum, the expression levels of *BEL1* and *TFL1* were much higher in the axillary bud of the *phyb* mutant, which showed a restricted shoot branching phenotype, than those of the wild-type^62^. Recent research showed that overexpression of *JcTFL1b* decreased shoot branching capacity in *J. curcas* ^63, 64^. Here, our results showed that the expressions of *JcBEL1* (Cluster-26.17792) and *JcTFL1b* (Cluster-26.17723) in *J. curcas* were significantly decreased in the buds after GA_3_ or BA treatment (Fig. 3C and J), as in the activated axillary buds of hybrid aspen, where *TFL1* ortholog *CENL1* is down-regulated during axillary bud activation^65^, suggesting that down-regulation of *JcBEL1* and *JcTFL1b* was correlated with bud outgrowth in *J. curcas. Dormancy-associated protein1* (*DRM1*) is associated with bud para-dormancy and is routinely used as a marker for bud burst^37, 66^. An inverse correlation between the expression level of *DRM1* and bud outgrowth was also found in many other plants, such as *Arabidopsis* ^51^, kiwifruit^66^, pea^67^ and sorghum^62^. Our results showed that GA_3_ or BA treatment of the axillary bud significantly decreased the expression levels of *JcDRM1* (Cluster-26.5833) and *JcDRM1-LIKE* (*JcDRML*, Cluster-26.12832) (Fig. 3B and E and Table S5). The strong inverse correlation between the *JcDRM1* and *JcDRML* expression levels and bud outgrowth indicated that these genes could be involved in the regulation of bud dormancy in *J. curcas*; nevertheless, the precise regulatory function of the *JcDRM1* and *JcDRML* remains unclear.

### GA3, BA and SL antagonistically regulate NAM/ATAF1/CUC2 (NAC) family genes

**Figure 5:**
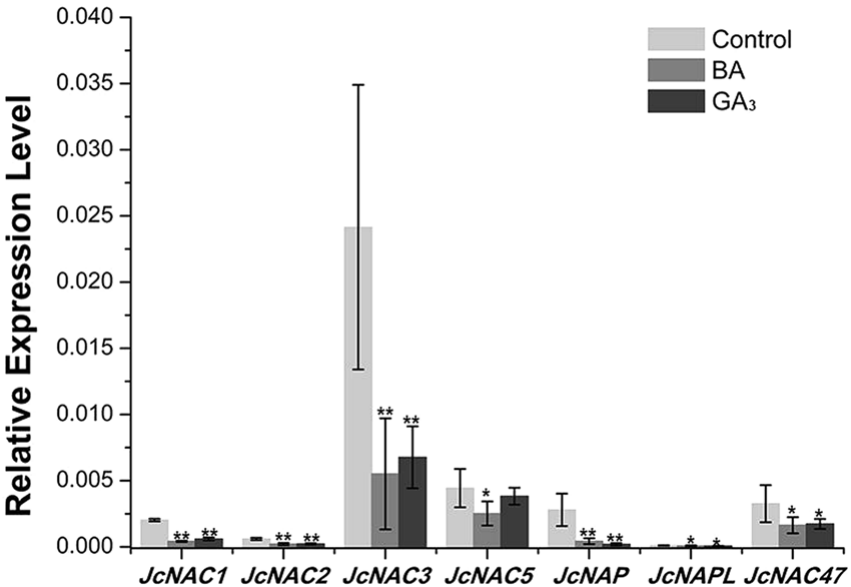
Quantitative real-time PCR analysis of a group of *NAC* genes negatively regulated by both GA_3_ and BA treatment. Cluster IDs: *JcNAC1*, Cluster-26.19073; *JcNAC2*, Cluster-26.5606; *JcNAC3*, Cluster-26.6331; *JcNAC5*, Cluster-26.20575; *JcNAP*, Cluster-26.21633; *JcNAPL*, Cluster-26.1094; *JcNAC47*, Cluster-26.19991. *GAPDH* was used as the internal reference. The error bars represent SE (n = 3). Student’s t-test was used to determine significant differences between the indicated and control groups. Significance levels: *P < 0.05; **P < 0.01.

*NAC* family genes are plant-specific and involved in many types of developmental, morphogenic and biotic or abiotic systems^68–70^. The *NAC* gene family has many members; however, only a few of these genes have been well characterized. Several *NAC* genes have been studied for their crucial role in the shoot apical meristem and floral organs^71–75^. Other developmental processes have also been shown to involve the *NAC* genes, such as axillary root development^76^, cell division^77^, embryogenesis^78^, programmed cell death (PCD)^79, 80^ and secondary cell wall formation^81^. In *J. curcas*, approximately 100 *NAC* genes were previously identified^82^. The results of the present study showed that a group of *NAC* genes, including *JcNAC1* (Cluster-26.19073), *JcNAC2* (Cluster-26.5606), *JcNAC3* (Cluster-26.6331), *JcNAC5* (Cluster-26.20575), *JcNAC47* (Cluster-26.19991), *JcNAP* (Cluster-26.21633) and *JcNAPL* (Cluster-26.1094), were down-regulated by both GA_3_ and BA treatment among the 250 co-regulated genes (Fig. 5 and Table S5). Considering the biological functions of the *NAC* genes in the regulation of shoot apical meristem and axillary meristem development, we hypothesized that these co-regulated *NAC* genes were involved in the regulation of bud outgrowth by GA_3_ or CK in *J. curcas*.

**Figure 6:**
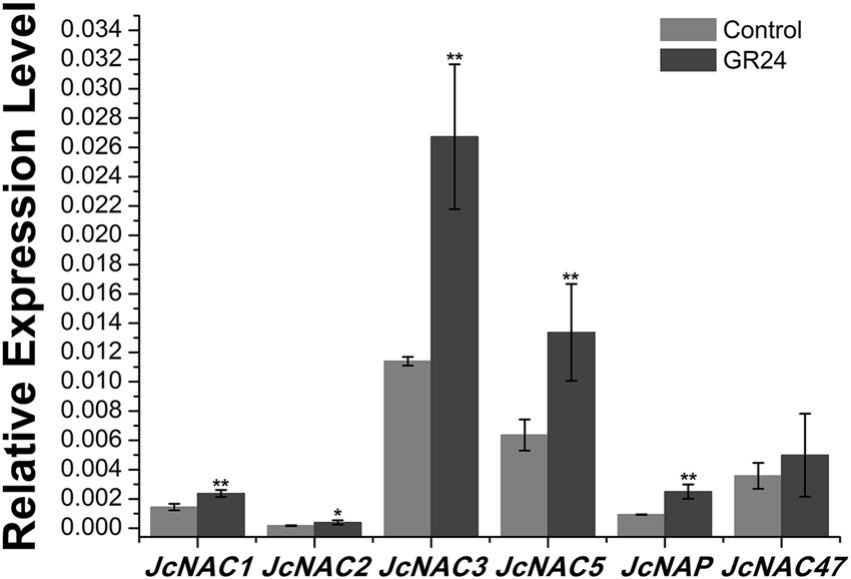
SL up-regulates the expression of a group of *NAC* genes at the axillary buds at 12 h. Cluster IDs: *JcNAC1*, Cluster-26.19073; *JcNAC2*, Cluster-26.5606; *JcNAC3*, Cluster-26.6331; *JcNAC5*, Cluster-26.20575; *JcNAP*, Cluster-26.21633; *JcNAC47*, Cluster-26.19991. *GAPDH* was used as the internal reference. The error bars represent SE (n = 3). Student’s t-test was used to determine significant differences between the indicated and control groups. Significance levels: *P < 0.05; **P < 0.01.

SLs are inhibitors of axillary bud outgrowth in pea^10, 11^ and *J. curcas* ^19^. Intriguingly, in *J*. *curcas*, GR24 (a synthetic analog of SL) treatment significantly up-regulated the expression of *NAC* genes, in contrast to the regulation by GA_3_ or BA (Fig. 6). This contrasting regulation of *NAC* genes is consistent with the physiological results showing that SL inhibited the bud outgrowth promoted by GA_3_ or BA treatment^19^, which further suggested that these co-regulated *NAC* genes may be involved in the regulation of bud outgrowth in response to phytohormones.

### BA regulates GA biosynthesis, catabolism and signaling genes in the buds

**Figure 7:**
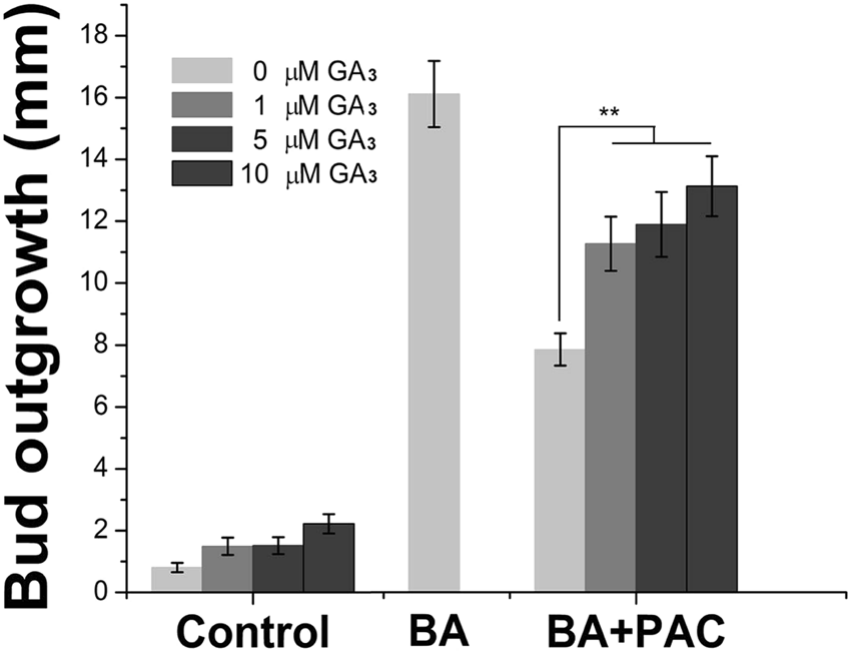
GA_3_ decreases the PAC inhibition of bud outgrowth induced by BA treatment. Low concentrations of GA_3_ (1, 5 and 10 μM) were co-applied with the BA + PAC solution (200 μM each). The error bars represent SE (n > 20). Student’s t-test was used to determine the significant differences between the indicated and control groups. Significance levels: **P < 0.01.

In the previous study^19^, we demonstrated that GA_3_ was required for the promotion of bud outgrowth by BA, and GA_3_ and CK synergistically regulate the axillary bud outgrowth in *J. curcas*. In this study, we found that addition of GA_3_ significantly reduced the inhibition of bud outgrowth by the paclobutrazol (PAC), an inhibitor of GA biosynthesis, in the BA + PAC combined treatment (Fig. 7), demonstrating that GA_3_ was directly involved in the promotion of bud outgrowth by CK.

The concentration of bioactive GAs is regulated by the expression of genes encoding GA 20-oxidases (GA20ox) and GA 3-oxidases (GA3ox), responsible for the late steps of GA biosynthesis, as well as GA 2-oxidases (GA2ox), responsible for GA degradation^83, 84^. The transcriptome analysis showed that BA significantly increased the expression of *JcGA20ox2* (Cluster-26.16883), and down-regulated the expression of *JcGA2ox2* (Cluster-26.9482) and *JcGA2ox4* (Cluster-26.18818) (Table S5), indicating that BA could promote GA biosynthesis and inhibit GA degradation in the buds of *J*. *curcas*.

We further analyzed the expression patterns of whole gene families involved in GA biosynthesis (principally *JcGA20ox*s and *JcGA3ox*s) and degradation (*JcGA2ox*s) using qPCR in the young axillary buds after BA treatment. Based on *J*. *curcas* genome sequences^30, 31^, we identified two *JcGA20ox*, three *JcGA3ox*, and five *JcGA2ox* gene members. The qPCR analysis revealed that the expressions of *JcGA20ox* and *JcGA3ox1*, which is the major member of *JcGA3ox* gene family expressed in the young axillary buds, were up-regulated after BA treatment; while all five members of *JcGA2ox* family were substantially down-regulated (Fig. 4), consistent with the transcriptome analysis results (Table S5). These results demonstrated that, at least in the young axillary buds, CK can promote GA accumulation and cooperatively regulate bud outgrowth. And the fact that BA had a relatively stronger effect on the promotion of axillary bud outgrowth^19^ likely reflects the combined effects of BA itself and the increased endogenous GA level induced by BA, rather than the effect of BA alone. This is in contrast with the results in *Arabidopsis*, where CK promotes GA degradation by inducing the GA deactivation gene *AtGA2ox2* expression^85^.

**Figure 8:**
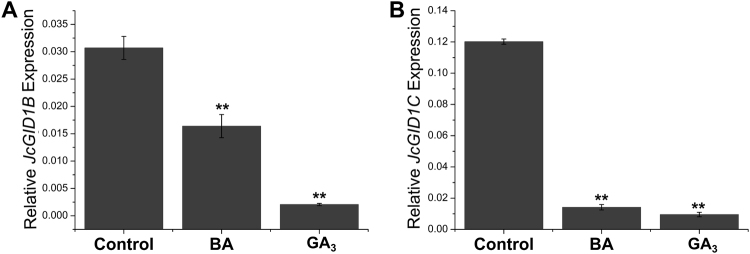
BA or GA_3_ treatment inhibits the expression of GA receptor genes, *JcGID1B* (**A**, Cluster-26.2964) and *JcGID1C* (**B**, Cluster-26.6734). *GAPDH* was used as the internal reference. The error bars represent SE (n = 3). Student’s t-test was used to determine the significant differences between the indicated and control groups. Significance levels: **P < 0.01.

In addition, two genes encoding GA receptors, *JcGID1B* (Cluster-26.2964) and *JcGID1C* (Cluster-26.6734), were significantly down-regulated after BA or GA_3_ treatment in the buds (Fig. 8 and Table S5). Since BA treatment promoted GA biosynthesis and inhibited GA degradation in the buds, the down-regulation of GA receptors *JcGID1s* expression in response to both BA and GA_3_ treatment likely reflects the feedback regulation of GA signaling as shown in *Arabidopsis* ^86^. Similarly, GA biosynthesis genes (*GA20ox*s and *GA3ox*s) were significantly down-regulated, while GA degradation genes *JcGA2ox2* and *JcGA2ox4* were up-regulated after GA_3_ treatment (Fig. S4 and Table S5), indicating exogenously applied GA_3_ inhibited endogenous GA biosynthesis in *J*. *curcas*, which is consistent with observations in other plants^87^.

On the other hand, the transcriptome analysis showed that GA_3_ significantly down-regulated the expression of *adenosine phosphate isopentenyl transferase 3* (*JcIPT3*, Cluster-2015.0) and *Lonely Guy 1* (*JcLOG1*, Cluster-26.20104) (Table S3), both of which encode key enzymes involved in CK biosynthesis^88^. This result was consistent with a previous study showing that GA_3_ negatively regulated CK biosynthesis at the young axillary buds via inhibition of *IPT* family genes^19^, suggesting that the bud outgrowth induced by GA_3_ treatment is not through regulation of CK biosynthesis in the young axillary buds.

It is noteworthy that the GA_3_, one of the bioactive GAs, was used to study the transcriptome response of young axillary buds to GA in *J. curcas* in this report. However, Rinne *et al*. found that GA_4_ induced canonical bud burst and development in a concentration-dependent way, while GA_3_ failed to induce the same response in *Populus* ^48^. And Eriksson *et al*. found that GA_4_ is the active GA in the regulation of *Arabidopsis* floral initiation^89^. Therefore, further studies will be needed to identify which GAs are responsible for the regulation of shoot branching in *J. curcas*, although gibberellins GA_3_, GA_4_, and GA_4+7_ were all similar in their ability to stimulate branching from lateral buds in sweet cherry^20^.

## Conclusion

Phytohormones are the major determinants in controlling axillary bud outgrowth. The identification of the hormone-responsive genes that are associated with bud dormancy and outgrowth could provide insight into the regulation of axillary bud growth. RNA-seq transcriptome expression analysis was used to elucidate the mechanisms underlying bud outgrowth control and identify the potential genes involved in the regulation of bud outgrowth. Both BA and GA_3_ could directly promote bud outgrowth in *J*. *curcas*. To further elucidate how the axillary bud responds to hormones and regulatory pathways in *J. curcas*, we identified the DEGs after GA_3_ or BA treatment using transcriptome sequencing. Overall, 250 DEGs (fold-change ≥ 2, FDR < 0.05) were co-regulated in response to GA_3_ or BA treatment and also exhibited similar expression patterns. These results suggested that GA_3_ and BA synergistically regulated axillary bud outgrowth in *J*. *curcas*, which is consistent with the physiological results. Moreover, BA treatment may promote GA accumulation in the young axillary buds as a result of the up-regulation of GA biosynthesis genes and the down-regulation of GA degradation genes in the axillary bud. This study helped to elucidate the cooperative relationship between these two hormones in regulating axillary bud outgrowth in *J*. *curcas*.

## Materials and Methods

### Plant materials and growth conditions

The *J. curcas* cultivar ‘Flowery’ was used in all experiments. Seedlings were planted in plastic growth pots (300 mL) under long-day photoperiod (14 h light/10 h dark). The growth temperature was 25 °C, and the light intensity was approximately 100 mol m^−2^ s^−1^. Twenty pots were placed in a plastic pallet. The seedlings were irrigated every other day by directly adding 1 L of distilled water into the pallet and fertilized with 1/4 MS containing only macroelements and microelements every other week as previously reported^19^.

### Hormone treatment

Initially, the hormones were prepared as stock solutions (20 mM) and stored at −20 °C. GA_3_ and BA were separately dissolved in water-free ethanol and 0.3 M NaOH. The working solutions were prepared using the stock solutions. Each working solution (mock, GA_3_ and BA) contained the same concentrations of ethanol, NaOH and Tween-20 as reported previously^19^. Paclobutrazol (PAC) and GR24 were separately dissolved in methanol and acetone to obtain a stock solution of 10 mM, and then diluted with water to the final working concentrations. Controls were treated with distilled water containing the same concentrations of methanol or acetone and Tween-20 as reported previously^19^. For hormone treatments, 20 μL of the working solutions was directly dropped onto the leaf axil at node 1 of the three-week-old *J. curcas* seedlings. Twelve hours after hormone treatment, the node stem (approximately 10–20 mg, 3–4 mm by length) at node 1, where the axillary bud located, was sliced and immediately frozen in liquid nitrogen for RNA isolation and transcriptome sequencing analysis.

### RNA isolation and sequencing

For each treatment, three biological replicates were prepared from a pool of axillary buds from approximately 20 three-week-old *Jatropha* seedlings. RNA isolation was performed using the Biozol Plant RNA Extraction Kit, as previously described^19^. RNA quantity and quality were assessed using a NanoDrop 2000c Spectrophotometer (Thermo Scientific, Wilmington, DE, USA) and agarose gel electrophoresis, respectively. The RNA samples were further assessed using the Agilent Bioanalyzer 2100 system (Agilent Technologies, CA, USA). The mRNA was enriched from total RNA using oligo (dT) magnetic beads and fragmented into approximately 200-bp fragments. The cDNA was synthesized using a random hexamer primer and purified with magnetic beads. After the end reparation and 3′-end single nucleotide acid addition, the adaptors were ligated to the fragments. The fragments were enriched through PCR amplification and purified using magnetic beads. The libraries were assessed using the Agilent 2100 Bioanalyzer and quantified using the ABI StepOnePlus Real-Time PCR System. The samples were sequenced with single-end on an Illumina HiSeq^TM^ 2000 (BGI Tech, Shenzhen, China).

### De novo assembly of transcriptome and abundance estimation

Low-quality reads with Phred scores < 20 were trimmed using Fastq_clean^90^, and the data quality was assessed using FASTQC^91^. The filtered reads were assembled using Trinity (version 2.0.6) with default parameters^92, 93^. The filtered reads from each library were mapped to de novo assemblies using Bowtie version v1.1.1 by allowing two mismatches (-v 2 -m 10)^94^. The transcript abundance was estimated using Corset (version 1.03)^95^. The count data generated from Corset were processed using the edgeR package^44^. Transcripts with less than one count per million reads (CPM) for at least three libraries were removed, and the remaining data were used for the next analysis. A matrix was constructed using the single factor style. Effective library sizes were determined using the trimmed mean of M values (TMM) normalization method. The common dispersion and tag wise dispersion were estimated using the quantile-adjusted conditional maximum likelihood (qCML) method. A multidimensional scaling was performed through the “plotMDS.dge” function of the edgeR package^44^. The exact test was performed to compute the expression of genes between the treatment and mock groups. Raw P values were adjusted for multiple testing using a false discovery rate (FDR)^96^. Genes with an FDR ≤ 0.05 and fold change (FC) ≥ 2 were regarded as differentially expressed genes (DEGs). GO analysis of the DEGs and pathways were processed using the DAVID^97^ with a cutoff of P-value ≤ 0.01. Hierarchical clustering of the co-regulated genes listed in Table S5 was performed using the pheatmap R package (version 1.0.7)^98^.

### Quantitative real time PCR analysis (qRT-PCR)

For each sample, 1 μg RNA was used for cDNA synthesis using the PrimeScript Kit (TaKaRa Biotechnology, Dalian, China). qPCR was performed using TaKaRa SYBR Premix Ex Taq^TM^ II (TaKaRa Biotechnology, Dalian, China). Quantitative real-time PCR was performed on three independent biological replicates for each sample and three technical replicates, using the Roche Light Cycler 480II System (Roche, Swiss). The primers used in the qRT-PCR analysis are listed in Table S10.

### Availability of supporting data

All RNA-seq data obtained in the present study are listed in GenBank under accession number SRP048608. The GenBank accession numbers of the DEGs described in the paper are listed in Table S11. The full length sequences of the DEGs by GA_3_ and BA treatment are listed in Table S12.

## Electronic supplementary material

Supplementary information

Dataset 1

Dataset 2

Dataset 3

Dataset 4

Dataset 5

Dataset 6

Dataset 7

Dataset 8

Dataset 9

Dataset 10
