# Supplementary material for: Comparative transcriptome analysis of axillary buds in response to the shoot branching regulators gibberellin A3 and 6-benzyladenine in Jatropha curcas
Source: Sci Rep. 2017 Sep 12;7:11417. doi: 10.1038/s41598-017-11588-0 (PMC5595854; doi:10.1038/s41598-017-11588-0)
Supplement: Supplementary file 1 — Supplementary information [file 41598_2017_11588_MOESM1_ESM.doc]

**Supplementary information**

**Comparative transcriptome analysis of axillary buds in response to the shoot branching regulators gibberellin A3 and 6-benzyladenine in *Jatropha curcas***

Jun Ni^1, 2, †^ [nijun@ipp.ac.cn](mailto:nijun@ipp.ac.cn)

Mei-Li Zhao^1, 3, †^ [1373825120@qq.com](mailto:1373825120@qq.com)

Mao-Sheng Chen^1^ [chenms@xtbg.org.cn](mailto:chenms@xtbg.org.cn)

Bang-Zhen Pan^1^ [pbz@xtbg.org.cn](mailto:pbz@xtbg.org.cn)

Yan-Bin Tao^1^ taoyanbin@xtbg.ac.cn

Zeng-Fu Xu^1^, * [zfxu@xtbg.ac.cn](mailto:zfxu@xtbg.ac.cn)

^1^ Key Laboratory of Tropical Plant Resources and Sustainable Use, Xishuangbanna Tropical Botanical Garden, Chinese Academy of Sciences, Menglun, Mengla, Yunnan 666303, China

^2^ Institute of Technical Biology & Agriculture Engineering, Hefei Institutes of Physical Science, Chinese Academy of Sciences, Hefei, Anhui 230031, China

^3^ College of Life Sciences, University of Chinese Academy of Sciences, Beijing 100049, China

† Authors contribute equally to this article.

* Corresponding Author: Z.-F. Xu (email: zfxu@xtbg.ac.cn)

##
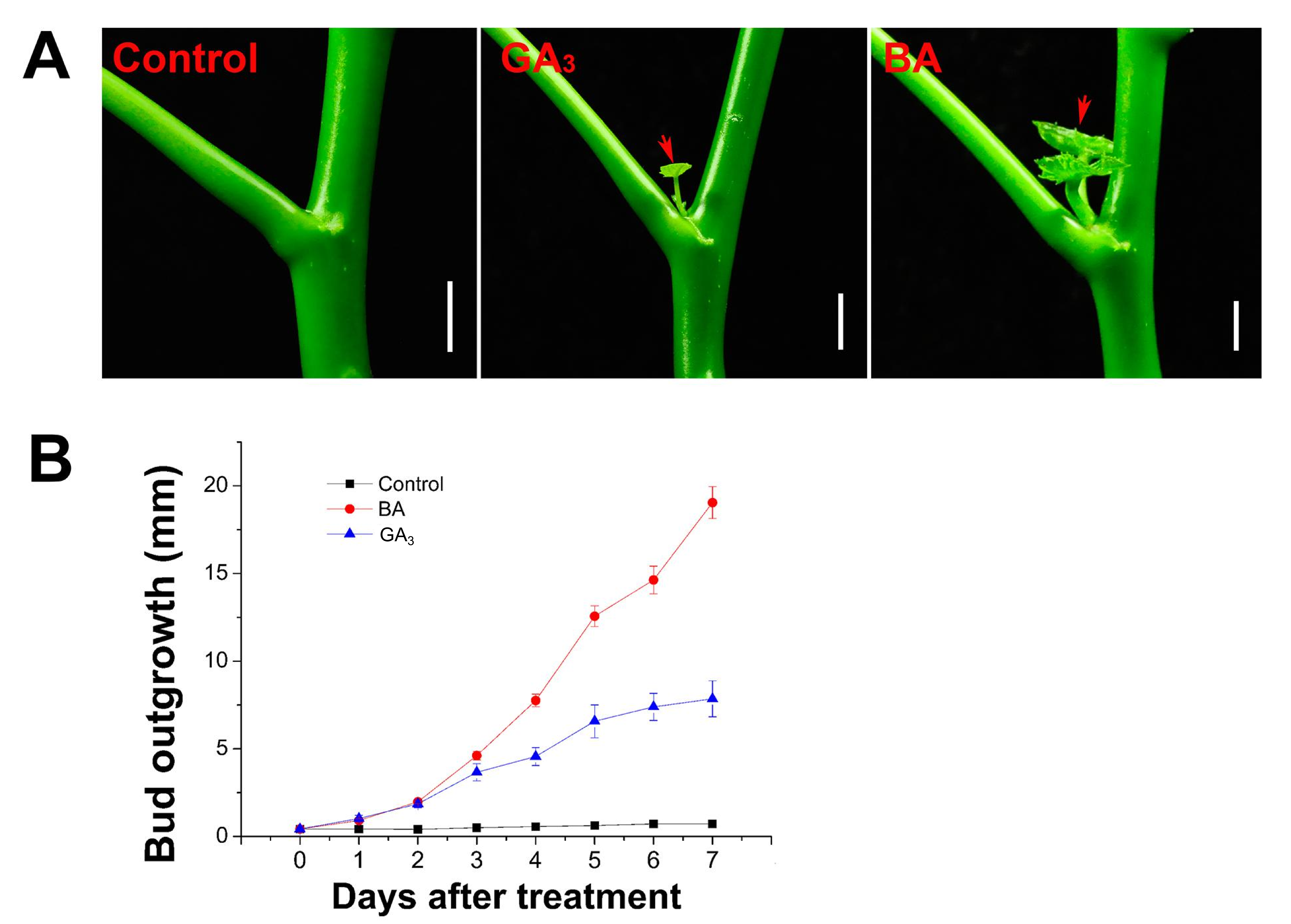


## Fig. S1 GA_3_ and BA promote axillary bud outgrowth at node 1 of the three-week-old *J. curcas* seedlings. (A) Photographs of the bud outgrowth at node 1 one week after GA_3_ or BA treatment. (B) Bud length at node 1 was measured from day 1 to day 7 (n > 20). The concentrations of GA_3_ and BA were both 500 μM. Bars = 1.0 cm.

**
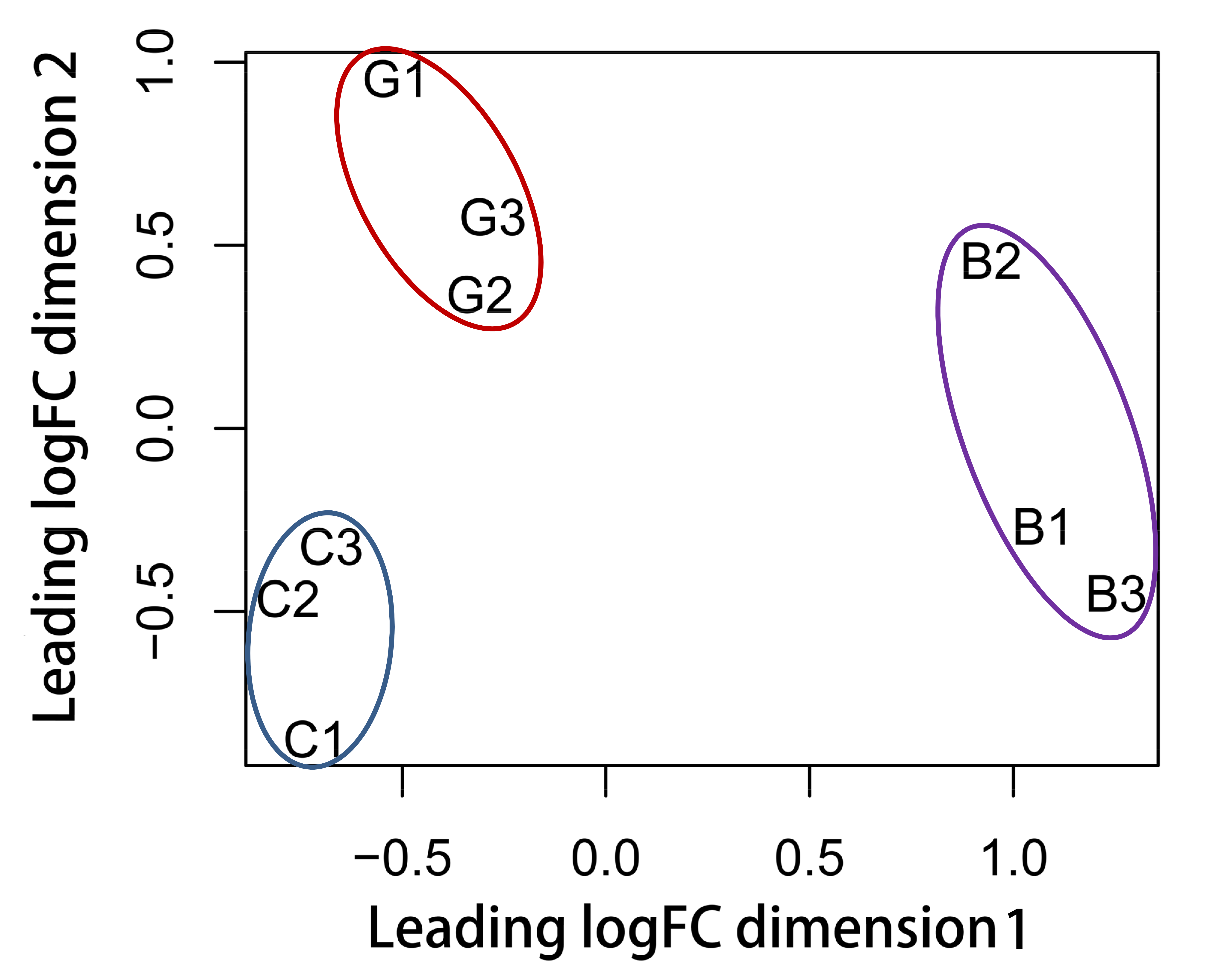
**

**Fig. S2** **Multidimensional scaling (MDS)** **plots showing the distance among nine samples.** C1, C2 and C3 indicate mock treatment. B1, B2 and B3 indicate BA treatment. G1, G2 and G3 indicate GA_3_ treatment. logFC: log_2_ (fold change).


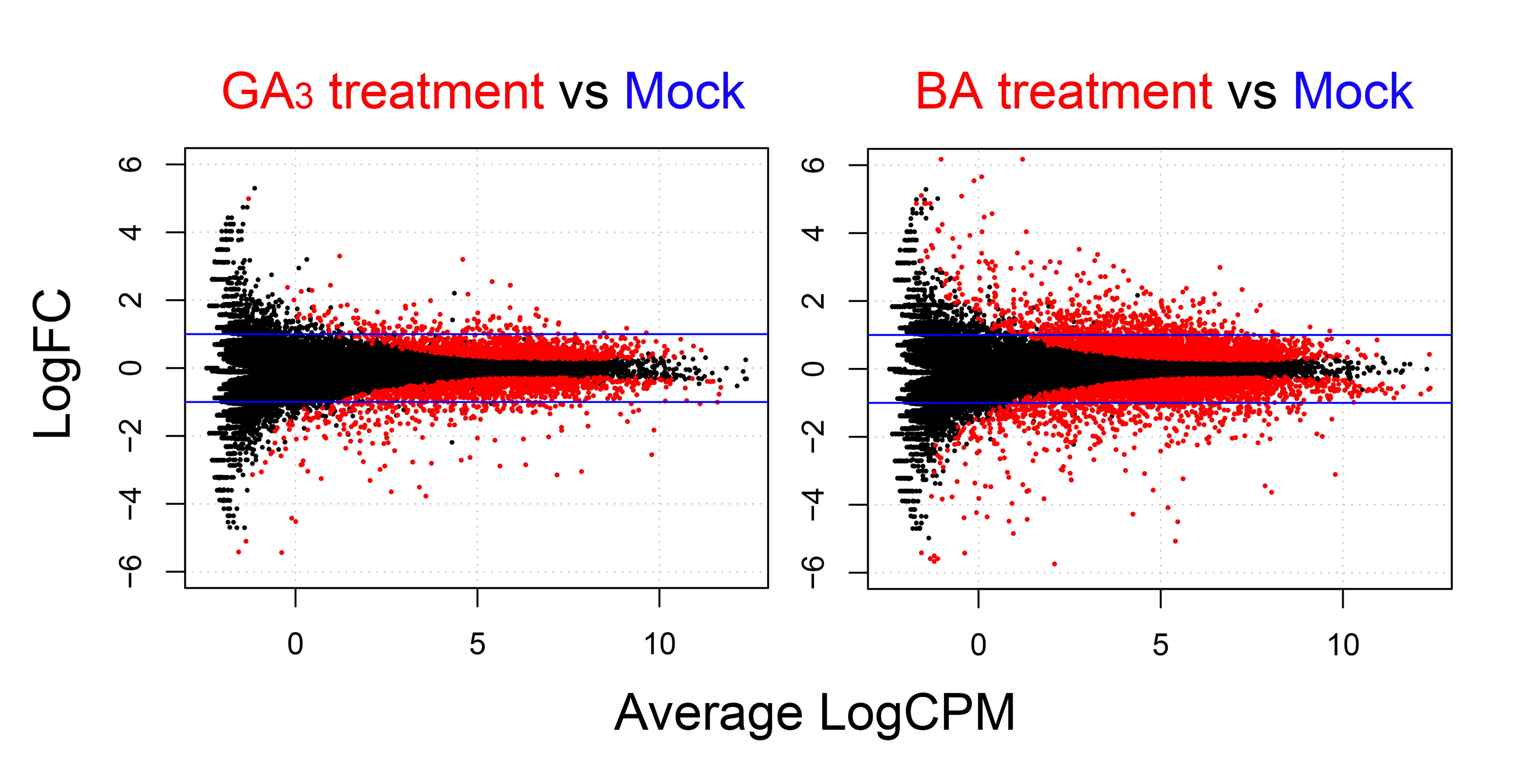


**Fig. S3** **Pairwise comparisons of gene expression between** **GA_3_, BA and mock samples.** Blue lines indicate the two-fold changed genes. Red points indicate differentially expressed genes (DEGs) with a false discovery rate (FDR) of <0.05. FC, fold-change. CPM, counts per million mapped reads.

**
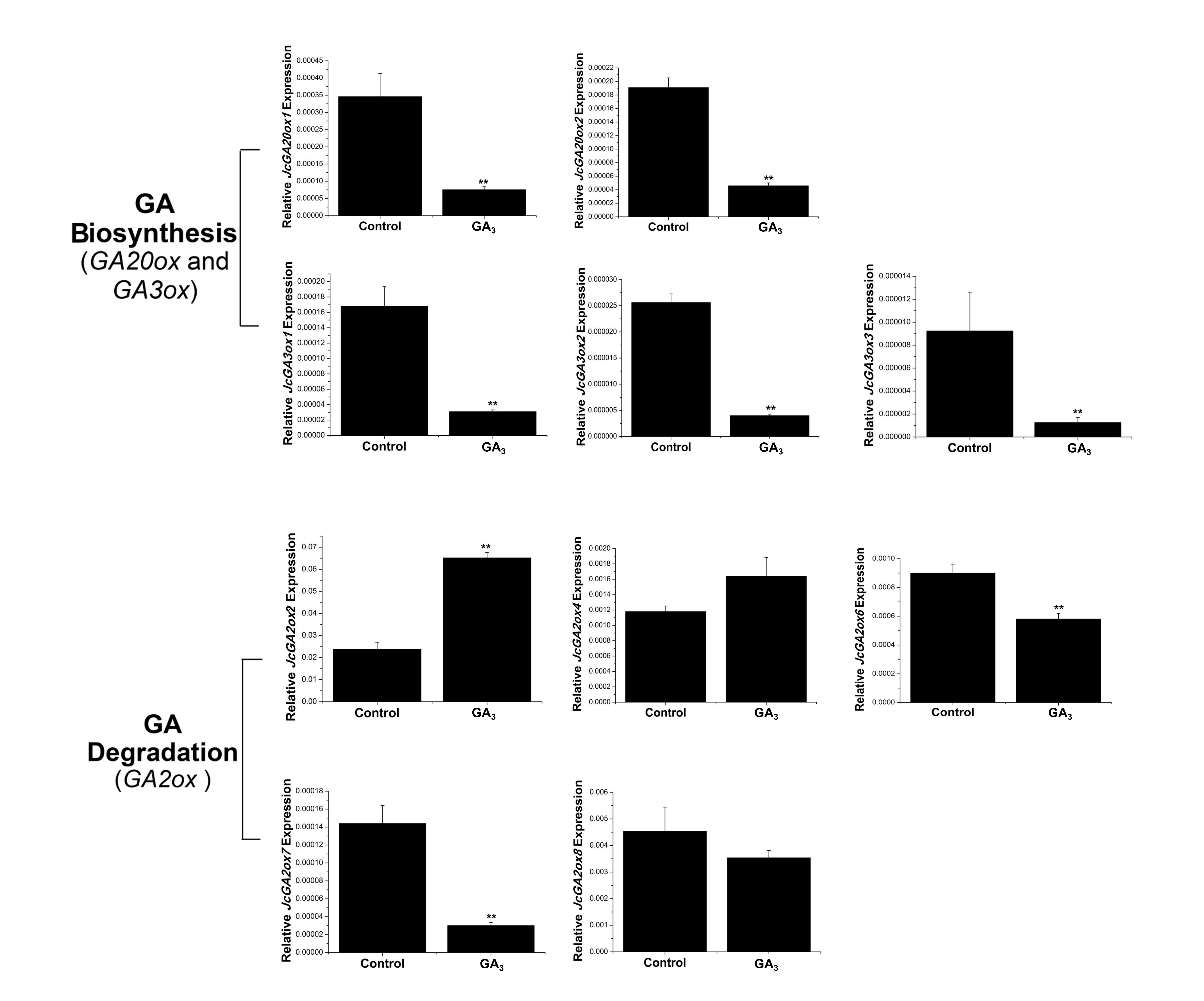
**

**Fig. S4 GA biosynthesis and degradation genes regulated by GA_3_ treatment.** GA_3_ treatment inhibited the expression of the GA biosynthesis genes *JcGA3ox*s and *JcGA20ox*s. It also promoted some of the GA degradation genes, while promoted the others in *Jatropha curcas*. Cluster IDs: *JcGA20ox1*, Cluster-26.319; *JcGA20ox2*, Cluster-26.16883; *JcGA3ox1*, Cluster-26.16438; *JcGA3ox2*, Cluster-26.14433; *JcGA3ox3*, Cluster-26.14518; *JcGA2ox2*, Cluster-26.9482; *JcGA2ox4*, Cluster-26.18818; *JcGA2ox6*, Cluster-26.14996; *JcGA2ox7*, Cluster-26.16004; *JcGA2ox8*, Cluster-26.18801. GAPDH was used as the internal reference. The error bars represent SE (n = 3). Student’s t-test was used to determine the significant differences between the indicated and control groups. Significance levels: ** P<0.01.

**Table S1 Sequencing read counts and alignment statistics for the sequenced *Jatropha curcas* samples.**

| **Sample** | **Treatment** | **Clean reads** | **Q30 (%)** | **% Mapped^*^** |
| --- | --- | --- | --- | --- |
| C1 | mock | 11,801,533 | 98.48 | 97.83 |
| C2 | mock | 11,197,651 | 95.37 | 97.69 |
| C3 | mock | 12,088006 | 97.07 | 97.88 |
| G1 | GA_3_ | 11,212,820 | 93.50 | 97.97 |
| G2 | GA_3_ | 11,531,837 | 92.25 | 97.98 |
| G3 | GA_3_ | 11,491,383 | 95.27 | 98.08 |
| B1 | BA | 11,610,300 | 95.19 | 97.98 |
| B2 | BA | 12,460,895 | 95.90 | 97.93 |
| B3 | BA | 11,857,474 | 97.00 | 98.14 |

^*^ Mapped read indicates the aligned reads.

**Table S2** **List of DEGs in the BA treated samples.**

**Table S3** **List of DEGs in the GA_3_ treated samples.**

**Table S4** **GO analysis of the DEGs by GA_3_ or BA treatment.**

**Table S5** **List of the co-regulated DEGs by GA_3_ and BA treatment.**

**Table S6** **List of the BA-specific DEGs.**

**Table S7** **List of the GA_3_-specific DEGs.**

**Table S8** **GO analysis of 97 co-up-regulated DEGs by GA_3_ and BA treatment.**

**Table S9** **GO analyses of 138 co-down-regulated DEGs by GA_3_ and BA treatment.**

**Table S10** **List of primers used for qPCR analysis in this study.**

**Table S11 List of GenBank accession numbers of the DEGs.**

| **Gene name** | **Cluster ID** | **GenBank accession no.** |
| --- | --- | --- |
| *JcBEL1* | Cluster-26.17792 | XM_012231450.1 |
| *JcCDC45* | Cluster-26.14626 | XM_012229683.1 |
| *JcCDC6* | Cluster-2004.0 | XM_012212470.1 |
| *JcDEAR2* | Cluster-26.17120 | XM_012212919.1 |
| *JcDRM1* | Cluster-26.5833 | NM_001308768.1 |
| *JcDRM1-LIKE* | Cluster-26.12832 | KY244300 |
| *JcGA20ox1* | Cluster-26.319 | KM454465 |
| *JcGA20ox2* | Cluster-26.16883 | KM454471 |
| *JcGA2ox2* | Cluster-26.9482 | KDP37976 |
| *JcGA2ox4* | Cluster-26.18818 | KDP27967 |
| *JcGA2ox6* | Cluster-26.14996 | KDP28294 |
| *JcGA2ox7* | Cluster-26.16004 | KDP39055 |
| *JcGA2ox8* | Cluster-26.18801 | KDP30016 |
| *JcGA3ox1* | Cluster-26.16438 | KDP37169 |
| *JcGA3ox2* | Cluster-26.14433 | KDP32294 |
| *JcGA3ox3* | Cluster-26.14518 | KDP32333 |
| *JcGID1B* | Cluster-26.2964 | KY244301 |
| *JcGID1C* | Cluster-26.6734 | XM_012233749.1 |
| *JcGRF5* | Cluster-26.21078 | XM_012221642.1 |
| *JcICU2* | Cluster-761.0 | XM_012223857.1 |
| *JcIPT3* | Cluster-2015.0 | XM_012237097.1 |
| *JcLOG1* | Cluster-26.21058 | XM_012209406.1 |
| *JcNAC1* | Cluster-26.19073 | JF896576.1 |
| *JcNAC2* | Cluster-26.5606 | KM454462 |
| *JcNAC3* | Cluster-26.6331 | KM454463 |
| *JcNAC47* | Cluster-26.19991 | KM454464 |
| *JcNAC5* | Cluster-26.20575 | KC775279.1 |
| *JcNAP* | Cluster-26.21633 | KM454460 |
| *JcNAPL* | Cluster-26.1094 | KM454461 |
| *JcPDF1* | Cluster-26.15852 | XM_012211136.1 |
| *JcPOLA2* | Cluster-609.2 | XM_012230239.1 |
| *JcPrimPol* | Cluster-26.12318 | XM_012234784.1 |
| *JcTCP9* | Cluster-26.19926 | XM_012211148.1 |
| *JcTFL1b* | Cluster-26.17723 | KF944350.1 |
| *JcWRKY35* | Cluster-26.10011 | XM_012227515.1 |
| *JcWRKY47* | Cluster-1476.0 | XM_012227626.1 |

**Table S12 Full-length sequences of the DEGs by GA_3_ or BA treatment.**
